# An initial exploration of core collection construction and DNA fingerprinting in *Elymus sibiricus* L. using SNP markers

**DOI:** 10.3389/fpls.2025.1534085

**Published:** 2025-02-07

**Authors:** Xinrui Li, Daping Song, Mingfeng Li, Daxu Li, Minghong You, Yan Peng, Jiajun Yan, Shiqie Bai

**Affiliations:** ^1^ School of Life Science and Engineering, Southwest University of Science and Technology, Mianyang, China; ^2^ College of Grassland Science and Technology, Sichuan Agricultural University, Chengdu, China; ^3^ Institute of Herbaceous Plants, Sichuan Academy of Grassland Science, Chengdu, China

**Keywords:** *Elymus sibiricus* L., SNP, core collection, DNA fingerprinting, KASP, population identification

## Abstract

*Elymus sibiricus* L., an excellent forage and ecological restoration grass, plays a key role in grassland ecological construction and the sustainable development of animal husbandry. In China, the wild germplasm resources of *E. sibiricus* are abundant, and they are shaped by similar and contrasting climatic conditions to form distinct populations, which enrich the genetic diversity of *E. sibiricus*. To more comprehensively aggregate *E. sibiricus* germplasm resources at a lower cost and to more accurately utilize its genetic variation, this study conducted a preliminary exploration of core germplasm collections and fingerprinting of *E. sibiricus* using single nucleotide polymorphism (SNP) markers. By combining multiple evaluation measures with weighted processing, we successfully identified 36 materials from 90 wild *E. sibiricus* samples to serve as a core collection. Genetic diversity assessments, allele evaluations, and principal component analyses of the 36 core germplasm samples all indicate that these 36 samples accurately and comprehensively represent the genetic diversity of all 90 *E. sibiricus* germplasm accessions. Additionally, we identified 290 SNP loci from among the high-quality SNP loci generated by whole-genome sequencing of the 90 *E. sibiricus* samples as candidate markers. Of these, 52 SNP loci were selected as core markers for DNA fingerprinting of *E. sibiricus*. Using kompetitive allele-specific PCR (KASP) technology, we also performed population origin identification for 60 wild *E. sibiricus* germplasm accessions based on these core markers. The core SNP markers screened in this study were able to accurately distinguish between *E. sibiricus* germplasms from the Qinghai–Tibet Plateau and those from elsewhere. This study not only provides a reference for the continued collection and identification of *E. sibiricus* germplasm resources but also offers a scientific basis for their conservation and utilization.

## 1 Introduction

*Elymus sibiricus* L., the Elymus L. genus type species, is a perennial, self-pollinating, and allotetraploid grass widely distributed across Eurasia that plays a crucial role in meadow steppe and meadow communities, often as a dominant species (Yan et al., 2007b; Li et al., 2021). *Elymus sibiricus* has strong adaptability, and high forage quality. It naturally occurs across various habitats, including alpine meadows at elevations ranging from 1,500 to 4,900 meters, forest clearings, shrublands, slopes, and gravel beds along river valleys (Peng et al., 2022; Yen and Yang, 2022; Chen et al., 2023). *Elymus sibiricus* has abundant wild resources within China, where it mainly occurs in Northeast China, North China, Northwest China, and the Qinghai–Tibet Plateau (Li et al., 2021). *E. sibiricus* exhibits excellent cold resistance, with its seedlings able to withstand temperatures as low as -4°C, and it can safely overwinter under extreme low temperatures ranging from -40°C to -30°C (Yan et al., 2010). Chen and He (2004) found that even at an altitude of 3200 m in the Qinghai Lake region, *E. sibiricus* cv.duoye maintains high yields of forage and seeds. In North China, *E. sibiricus* typically returns to green in April, and in the Northwest region, it starts to germinate when the daily average temperature reaches above 0°C, and enters the greening period when the daily average temperature rises above 4°C (Li et al., 2000; Abulaiti et al., 2008). As a meso-xerophytic plant, *E. sibiricus* exhibits strong drought resistance during the germination and seedling stages, outperforming Agropyron cristatum (L.) Gaertn, and other congeneric species such as *E. dahuricus* Turcz., *E. nutans* Griseb., *E. excelsus* Turcz., and *E. tangutorum* (Nevski) Hand.-Maz in terms of drought tolerance (Yu et al., 2011; Chen et al., 2016; He et al., 2023). Liu et al. (2022) found that under drought stress, apoplastic barrier in the endodermis could maintain the balanced growth of *E. sibiricus*, which contributes to drought tolerance of *E. sibiricus*. The antioxidant defense system, key metabolic substances, specific transcription factor families, and genes related to signal transduction are also considered important factors in *E. sibiricus*’ strong drought resistance (Li et al., 2020; Yang et al., 2020; Yu et al., 2022; An et al., 2024). Additionally, *E. sibiricus* has a certain tolerance to salt stress, which makes it suitable for the restoration and improvement of saline-alkali lands (Yang et al., 2015). Based on these characteristics, *E. sibiricus* is extensively used for the revegetation of degraded grasslands, soil stabilization, and forage production in the Qinghai–Tibet Plateau and other high-altitude areas of western China (Xie et al., 2015; Bai and Yan, 2020). Thus far, *E. sibiricus* is among the few native grass species that have achieved large-scale seed production and commercial utilization in the Qinghai–Tibet Plateau region owing to its high seed yield potential (Yan et al., 2024). Previously, research on the germplasm resources of *E. sibiricus* has primarily focused on resource evaluation and genetic diversity assessment based on phenotypic characteristics and second-generation molecular markers such as simple sequence repeats (SSRs) and sequence-related amplified polymorphisms (SRAPs) (Ma et al., 2008; Yan et al., 2008; Zeng et al., 2022; Zhang et al., 2024). In contrast, Guo et al. (2016) used morphological traits and SSR markers to identify four *E. sibiricus* cultivars from the Northwest Plateau of Sichuan, China. Additionally, Xie et al. (2015) utilized start codon targeted polymorphisms (SCoT) markers to identify 69 different cultivars and wild accessions of *E. sibiricus*. However, there have been no reports identifying the population origins for wild *E. sibiricus* germplasm accessions. *E. sibiricus* germplasm from different habitats have experienced varying climate types, leading to significant phenotypic and genetic differences among wild eco-geographic populations, which provides rich genetic resources and diverse selection bases for *E. sibiricus* germplasm improvement (Zhang et al., 2022; Xiong et al., 2024b; Yan et al., 2024). Characterizing and analyzing wild *E. sibiricus* germplasm greatly facilitates the preservation and utilization of these resources. However, methods based on morphological traits and the use of second-generation molecular markers to identify the origins of wild *E. sibiricus* germplasm are both time-consuming and inefficient. Moreover, the morphological variation in wild *E. sibiricus* germplasm is extensive, and its traits are often influenced by environmental changes, dramatically increasing the difficulty of source identification and hindering the collection and utilization of these germplasm resources (Yan et al., 2007a; Li et al., 2023a). Therefore, establishing an economical, efficient, and accurate method for identifying the population source of *E. sibiricus* germplasm is crucial for advancing the research and conservation of its genetic resources, origin identification, and sustainable development of the *E. sibiricus* industry.

Recently, a high-quality *E. sibiricus* reference genome (6.53 GB) of ‘Chuancao No. 2’, a nationally approved variety in China, has been released, which provides a foundation for population genomics studies of *E. sibiricus*. Yan et al. (2024) performed whole-genome resequencing of 90 wild *E. sibiricus* germplasm samples from various habitat types and thus identified 80,148,422 high-quality SNP loci by alignment and comparison with the ‘Chuancao No. 2’ *E. sibiricus* reference genome. Based on these SNP loci, the study not only highlighted the rich genetic diversity of wild *E. sibiricus* germplasm but also was able to divide the 90 wild *E. sibiricus* germplasm samples into four distinct groups, Qinghai–Tibet Plateau (QTP), Northwest China (NW), North China (NC), and Northeast China (NE) groups, providing important genetic evidence for the population classification of *E. sibiricus* resources. Compared with molecular markers such as SSRs, inter-simple sequence repeat (ISSRs), and SRAPs, SNP markers are distributed more evenly across the entire genome, and they are characterized by higher density, greater polymorphism, and more stable inheritance (Rafalski, 2002; Varshney et al., 2009; Li et al., 2023c). SNPs can be readily adapted to automated genotyping methods and can be identified via high-throughput automated detection (Zhang et al., 2020). The KBiosciences Kompetitive allele-specific PCR (KASPar) system is one of the most ideal high-throughput SNP genotyping platforms given its high accuracy and low cost (Semagn et al., 2014), and it has been widely utilized in studies on wheat (Grewal et al., 2020), rice (Steele et al., 2024), cotton (Islam et al., 2015), cucumber (Kahveci et al., 2021), and broccoli (Shen et al., 2020, 2021). Thus, constructing a core collection based on SNP markers is more accurate and effective (Van Inghelandt et al., 2010; Dou et al., 2023). A core collection is characterized by its heterogeneity, diversity, representativeness, and practicality, as it is not merely a simple molecular genetic snapshot of an entire species but rather a representative subset that retains most of the genetic information of the original broader germplasm (Brown, 1989; Gu et al., 2023). The construction of a core collection not only provides strong support for genetic breeding, conservation biology, and systematic studies, but also has high academic and practical value in promoting germplasm resource exchange and utilization as well as gene bank management more generally (Gautam et al., 2004; Gu et al., 2023; Aribi, 2024). Lee et al. (2020) selected 67 pumpkin accessions as a core collection from among 610 pumpkin (Cucurbita moschata) germplasm accessions based on 2,071 high-quality SNPs. Similarly, Ketema et al. (2020) identified 94 accessions as a core collection from among 357 Ethiopian cowpea (Vigna unguiculata [L.] Walp.) germplasm samples based on genetic diversity analysis using SNP markers. These core accessions captured the full genetic diversity of the 357 Ethiopian cowpea germplasm samples, providing an empirical foundation for the collection, conservation, and utilization of Ethiopian cowpea germplasm. Girma et al. (2020) selected 387 sorghum (Sorghum bicolor L.) accessions as a core collection from 1,628 sorghum germplasms based on SNP markers, providing important resources for subsequent sorghum breeding, genomic research, and genetic studies.

Additionally, SNP markers are one of the marker types recommended by the International Union for the Protection of New Varieties of Plants (UPOV) in the BMT Molecular Testing Guidelines for constructing DNA fingerprint databases (Button, 2008). DNA fingerprint markers consist of a small number of highly representative markers that can be used to distinguish between different individuals or groups within the same species, and because of their advantages of being convenient and enabling rapid identification, as well as their accurate and stable results, they are widely used in the study of crop germplasm resource diversity and in variety identification (Karihaloo, 2015; Luo et al., 2023). Fan et al. (2021) selected 24 SNP loci with high polymorphism information content and strong sequence conservation to construct a DNA fingerprint map for tea plant (Camellia sinensis [L.] O. Kuntze) varieties, and it was used to accurately distinguish all 103 tested tea plant germplasm samples. Tian et al. (2021) constructed an SNP-DNA fingerprint database containing more than 20,000 maize (Zea mays L.) samples based on 200 core SNP loci; this database can thus be used in variety authentication, purity determination, and the protection of plant variety rights. Wang et al. (2021a) used SNP markers to construct a DNA fingerprint map for 216 cigar tobacco (Nicotiana tabacum L.) germplasm resources, providing a scientific basis for the selection and identification of high-quality cigar tobacco germplasm resources. However, the application of SNPs in the study of *E. sibiricus* has primarily focused on molecular marker-assisted breeding and inferring the demographic history of populations. For instance, Zhang et al. (2019) employed specific-locus amplified fragment sequencing (SLAF-seq) technology to successfully construct a high-density genetic linkage map for *E. sibiricus* and identify QTLs and candidate genes associated with seed traits. Moreover, they successfully identified genes related to the adaptation of *E. sibiricus* to high-altitude climatic conditions and important agronomic traits through Genetic Selection and Genome-Wide Association Analysis (GWAS) (Zhang et al., 2022). Xiong et al. (2024b) based on SNP data from the pan-chloroplast genome, inferred that the ancestors of *E. sibiricus* originated from the Qinghai-Tibet Plateau and underwent a complex migration history. Han et al. (2022) explored the factors influencing the geographic distribution pattern and genetic spatial structure of *E. sibiricus* on the Qinghai-Tibet Plateau using SNP markers. To date, there have been no reports on the construction of a core germplasm collection and DNA fingerprinting map for *E. sibiricus* based on SNP markers.

In this study, we thus constructed a core collection of *E. sibiricus* based on high-quality SNP markers generated from whole-genome resequencing of 90 wild *E. sibiricus* accessions (Yan et al., 2024). We further conducted genetic diversity analyses on these core accessions to evaluate their representativeness. Additionally, to accurately distinguish among different *E. sibiricus* germplasm sources and identify the population sources of wild germplasm accessions, we explored the construction of an *E. sibiricus* DNA fingerprint map based on SNP markers. Ultimately, a set of 52 core SNP markers was selected for the construction of the DNA fingerprint map. Subsequently, we utilized the KASPar platform to genotype 60 representative wild *E. sibiricus* accessions in order to differentiate them and identify their population sources, thereby validating the accuracy and effectiveness of the characterized core SNP markers. These findings not only enhance the conservation and utilization of *E. sibiricus* germplasm resources but also provide scientific evidence and data references for their continued collection and identification.

## 2 Materials and methods

### 2.1 Plant materials and DNA extraction

Nine *E. sibiricus* samples (**Supplementary Table 1**) were selected for Sanger sequencing to validate the accuracy of the core SNP loci. Sixty wild *E. sibiricus* samples (**Supplementary Table 2**) were also selected for KASP genotyping to evaluate the effectiveness of the 31 KASP markers. All plant materials were provided by the Sichuan Academy of Grassland Sciences (SAG). The seeds of these *E. sibiricus* samples were germinated in nutrient bowls (16 cm in diameter and 16 cm in height) filled with mixed soil (soil:nutrient soil:vermiculite, 3:4:1.5 [*V*:*V*:*V*]). The nutrient bowls were then placed in a growth chamber under controlled conditions (day/night cycle 16/8 h, 20/15°C; 60 ± 5% relative humidity; 400 µE·m−2 ·s −1 PPFD). For each germplasm accession, when the plants reached the seedling stage, young leaves were collected from each plant and stored at -80°C for subsequent DNA extraction.

The leaf samples were ground with approximately 4-mm-diameter steel beads in a Sceintz-48 tissue grinder (SCIENTZ, Ningbo, China) after sufficient chilling in liquid nitrogen. Genomic DNA was extracted from the plant samples using the TIANGEN Kit (TIANGEN BIOTECH, Co., Ltd., Beijing, China) according to the manufacturer’s instructions. The concentration and purity of the DNA were measured using a NanoDrop2000 UV spectrophotometer (Thermo Scientific, Waltham, MA, USA).

### 2.2 Data collection

The genomic data of *E. sibiricus* ‘Chuancao No. 2’ and the whole-genome sequencing data of 90 wild *E. sibiricus* samples (**Supplementary Table 1**) were downloaded from the National Genomics Data Center (NGDC) (BioProject accession number PRJCA029280). All SNP markers used in this analysis were derived from 80,148,422 high-quality SNP loci identified in the resequencing data of these 90 *E. sibiricus* germplasm samples (Yan et al., 2024).

### 2.3 Core collection development

To establish a core germplasm collection of *E. sibiricus* accessions, Core Hunter II software (Thachuk et al., 2009) was used to analyze and evaluate the representativeness of 90 *E. sibiricus* accessions. Using Core Hunter II software, we applied a weighted approach combining multiple evaluation measures (modified Roger’s distance and Shannon’s Diversity Index) to screen the number of core accessions for proportions of 0.1, 0.2, 0.3, 0.4, 0.5, 0.6, 0.7, 0.8, and 0.9 of the total germplasm collection, which was based on the parameters recommended on the Core Hunter website (https://github.com/cropinformatics/CoreHunter). The final core collection size was determined by evaluating allele coverage (CV) and comparing the expected heterozygosity (*H*_e_), Shannon–Weaver index, Nei’s gene diversity index, and polymorphism information content (PIC) between the core collection and the entire germplasm collection.

### 2.4 SNP selection

To select SNP markers with high marker quality, strong representativeness, high discriminative power, a uniform distribution across the genome, and high specificity for fingerprinting analysis, we followed five criteria (outlined below), based on the research of Sun (2017) and Wang et al. (2021a): (1) specificity, (2) uniform marker distribution and high marker quality, (3) substantial PIC values, (4) Hardy–Weinberg equilibrium, (5) uniqueness. (1) To ensure that the selected SNP markers have high specificity, we extracted 200 bp sequences upstream and downstream of each SNP, totaling 401 bp. We then performed BLAST alignments and retained only those SNPs that uniquely aligned to the reference genome. (2) Based on the premise that the SNP markers were uniformly distributed across the 14 chromosomes of *E. sibiricus*, we retained SNPs with no missing genotype data and discarded those with a minor allele frequency (MAF) below 20%. (3) The PIC values of SNP loci were calculated by a Perl script, and loci with PIC values less than 0.35 were discarded. (4) Hardy–Weinberg equilibrium was tested using VCFtools software (v.0.1.13) (Danecek et al., 2011) with the parameters –max-missing 1 –maf 0.2 –hwe 0.01, and the loci with a *p*-values greater than 0.01 were retained. (5) A Perl script was used to identify SNP loci that did not have mutations in other loci within 100 bp before and after labeling. Thus, we ultimately selected high-quality SNP markers for subsequent fingerprinting analysis.

### 2.5 Fingerprint construction and *generation* of 2D *barcodes*

The screened SNP loci were used to construct a DNA fingerprint map using RStudio (R version 4.4.0). To facilitate the viewing of genotype information for each germplasm accession, 2D barcodes were generated for each accession using the online software Caoliaoerweima (http://cli.im/), which can provide the genotype of each germplasm accession after the barcode is scanned.

### 2.6 Verification of SNP locus authenticity by sanger sequencing

The specific primers for 52 core SNP loci were designed using Primer Premier5 software (Lalitha, 2000). Parameters for primer design were as follows: GC content, 44%–72%, melting temperature (Tm), 56-68°C, and length, 19-25 bp, and the primers were synthesized by Sangon Bioengineering Co., Ltd. (Shanghai, China) (**Supplementary Table 3**). DNA from nine *E. sibiricus* samples, selected from the 90 materials, was used as the template for PCR amplification. The total volume of the PCR mixture was 20 μl, containing 1 μl Phanta Max Super-Fidelity DNA polymerase (Vazyme, Nanjing, China), 10 μl 2X Phanta Max buffer, 1 μl dNTP (10 mM each), 2 μL of genomic DNA, 0.8 μl of primer mix (containing 10 μM of primer F and 10 μM of primer R), and 5.2 μL of ddH_2_O. The PCR reaction conditions were as follows: initial denaturation at 94°C for 3 min, followed by 35 cycles of denaturation at 94°C for 30 s, annealing at 55°C for 30 s, and extension at 72°C for 1 min, with a final extension at 72°C for 5 min. All PCR-amplified products were separated by agarose gel electrophoresis, and the target fragments were extracted and recovered under a Tanon-3500 Gel Imaging System (Tanon Science & Technology Co., Ltd., Shanghai, China). The specific procedure is as follows: 5 ml of PCR product were subjected to 1% agarose gel electrophoresis at 150 V and 100 mA. After observing for 10-20 minutes, the target PCR band was excised from the gel, recovered. Subsequently, the target fragments were sequenced using the 3730XL sequencer (Thermo Fisher Scientific), and the sequencing data were analyzed using SnapGene (V6.0.2) (Li et al., 2023b) and SeqMan (v7) software (Swindell and Plasterer, 1997).

### 2.7 Kompetitive allele-specific PCR genotyping

For each SNP locus retained after screening, 200 bp sequences upstream and downstream of the SNP were extracted, and KASP primers were designed and developed by Genepioneer Biotechnologies (Nanjing, China). The online platform Primer3Plus (https://www.primer3plus.com/) was utilized for the design of KASP markers, wherein for each KASP target site, one common reverse primer and two allele-specific forward primers were designed based on the flanking sequences around the variant position (SNP). The primer design parameters are as follows: GC content between 30% and 60%, with an optimal GC content of 45%, Tm value between 55 and 61°C, and the size of the PCR product not larger than 120 bp. Primers were appended with tails compatible with the standard FAM or VIC labels (FAM tail: 5′-GAAGGTGACCAAGTTCATGCT-3′; VIC tail: 5′-GAAGGTCGGAGTCAACGGATT-3′), with a targeted SNP positioned at the 3′ end. Kompetitive Allele-Specific PCR (KASP) assays were performed using the CFX Connect™ Real-Time System (Bio-Rad, Hercules, CA, USA). The newly synthesized primers were diluted to 10 μM using TE buffer (pH 8.0) and mixed according to a ratio of forward genotyping primer 1:forward genotyping primer 2:reverse universal primer of 1:1:3 (*V*:*V*:*V*) to serve as the primer mix. DNA samples were diluted to match the concentration of the lowest concentration sample in the batch, and each 5-μL reaction mixture contained 1.25 μL of the diluted DNA sample. Finally, the total amplification reaction volume was 5 µL in each well of a 96-well plate and consisted of 2.5 µL of 2× KASP Master mix (JasonGen Biological Technology Co., Ltd, Beijing, China), 1.25 µL of primer mix, and 1.25 µL of DNA sample as described previously. The 96-well PCR reaction plates are subjected to sealing, shaking, and centrifugation to ensure thorough and even mixing of the reaction system. After centrifugation, the PCR reaction is carried out using the following cycling program: activation at 95°C for 10 min, 10 touchdown cycles of 95°C for 20 s and 61–55°C for 60 s (decreasing by 0.6°C each cycle), and then 27 cycles of denaturation and annealing/elongation at 95°C for 20 s and 55°C for 60 s. Fluorescent signals of the reactions were detected and genotyping data were analyzed using the Omega Fluorostar scanner (BMG Labtech, Ortenberg, Germany) and KlusterCaller (v2.22.0.5) software (Bansal et al., 2021).

### 2.8 Data analysis

Based on the population VCF file, we organized the genotyping information of selected SNP loci in 90 samples using the Notepad– (version 2.2) (https://gitee.com/cxasm/notepad–) text editor. Subsequently, the organized results were imported into PowerMarker (version 3.25) software to calculate genetic diversity indices, including Polymorphic Information Content (PIC), heterozygosity rate, Minor Allele Frequency (MAF), and Nei’s gene diversity index (Liu and Muse, 2005). Principal component analysis (PCA) was performed using Tassel (v5.1) (Bradbury et al., 2007), and based on the clustering results of the samples, the graphical representations were created using R language and ggplot2 package (Pryszcz and Gabaldón, 2016). The distance matrix between individuals was calculated based on SNPs using MEGA X (v10.2.6) (Kumar et al., 2018). The phylogenetic tree was constructed using IQ-TREE2 software (v2.2.0) based on the neighbor-joining method, with 1,000 bootstrap replicates (Minh et al., 2020). The specific parameter settings were as follows: -s *.phy –seqtype DNA -T 10 -B 1000 -m NJ –boot-trees. Based on the filtered SNPs, population structure was analyzed using Admixture software (v1.3.0) with the parameters -C 0.01 -s time –cv -j4 (Alexander et al., 2009). The number of subpopulations (K value) was pre-set to range from 1 to 10 for clustering, and cross-validation was performed on the clustering results. The optimal number of clusters was determined based on the minimum cross-validation error rate.

## 3 Result

### 3.1 Screening of core germplasm collection

**Figure 1:**
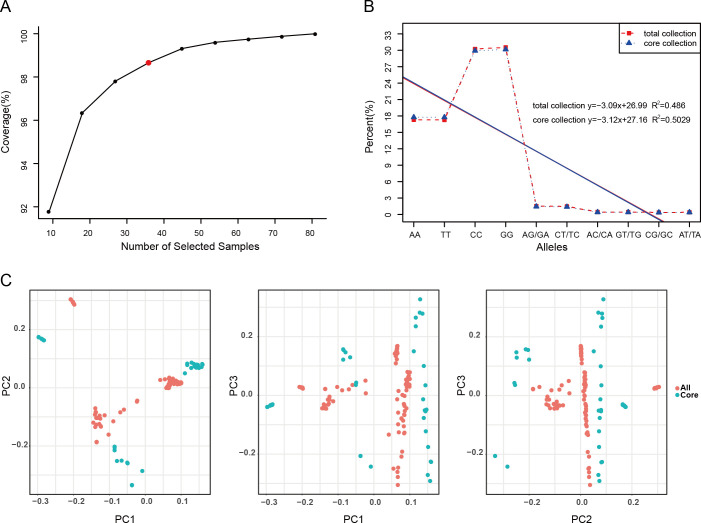
Screening and evaluation of the *Elymus sibiricus* core collection. **(A)** The evaluation of allele coverage for 90 *E. sibiricus* samples. The red dot in the graph represents the *E. sibiricus* core collection of 36 accessions. **(B)** The frequency distribution of genotypes in all 90 *E. sibiricus* samples and the core collection of 36 accessions. The ten potential genotypes are shown along the *x*-axis, and the *y*-axis shows the proportion of each genotype. The red dashed line indicates the genotype distribution of all germplasm resources, while the blue dashed line indicates the genotype distribution of the selected core germplasm accessions. The red solid line represents the fitted curve based on the genotype distribution of all germplasm resources, and the blue solid line represents the fitted curve based on the genotype distribution of the selected core germplasm. The upper right corner of the graph shows the linear equation fit to the data, and *R* ^2^ is the coefficient of determination. **(C)** The principal component analysis (PCA) plot of 90 *E. sibiricus* samples and the selected core collection. Each dot represents a sample, the red dots represent all 90 *E. sibiricus* samples, and the blue dots represent the core collection of 36 accessions.

In this study, a comprehensive evaluation and screening of 90 accessions of *E. sibiricus* germplasm resources were conducted according to weighted values of modified Roger’s distance (weight 0.7) and Shannon’s diversity index (weight 0.3). Through an assessment of coverage, 36 accessions (representing 40% of the germplasm resources) were ultimately identified as representative core germplasm accessions for *E. sibiricus* (**Figure 1A**; **Supplementary Table 4**, **Supplementary Data 1**). Further, we analyzed the genetic diversity of these 36 core accessions. The observed heterozygosity (*H*_o_) ranged from 0.028 to 1.000 (mean = 0.044), while the expected heterozygosity (*H*_e_) ranged from 0.095 to 0.500 (mean = 0.293). The Nei diversity index ranged from 0.097 to 0.509. Additionally, the average values of the Shannon–Wiener index and PIC were 0.459 and 0.243, respectively. These genetic diversity metrics of the core germplasm collection were found to be similar to those of the original full set of 90 accessions of *E. sibiricus* germplasm accessions (*P* < 0.05), indicating that the core germplasm collection effectively represents the overall genetic diversity (**Supplementary Table 5**). The allele frequency assessment revealed that the ten potential genotypes derived from the four nucleotides A, C, G, and T (as well as their dinucleotide combinations) were consistently distributed across all germplasm and the core germplasm (**Figure 1B**; **Supplementary Table 6**). Furthermore, the comparison of MAF values between the 36 core accessions and the entire germplasm pool indicated that the highest proportion of MAF values fell within the range of 0.05–0.10. The distribution of MAF values in the range of 0.20–0.50 was consistent between the core germplasm collection and the original full germplasm collection (**Supplementary Figure 1**). Finally, PCA of all germplasm materials and the core germplasm indicated that the core germplasm collection aligned well with the distribution plot of all *E*. *sibiricus* materials (**Figure 1C**). These results demonstrate that the 36 core accessions accurately capture the genetic diversity of all 90 *E. sibiricus* germplasm accessions.

### 3.2 SNP fingerprint construction

#### 3.2.1 The screening and evaluation of SNP markers

Based on resequencing data from 90 *E. sibiricus* germplasm accessions derived from four populations (NW, NE, NC, and QTP), with an average sequencing depth of approximately 11×, a total of 80,148,422 high-quality SNPs were identified for fingerprint profiling marker screening (Yan et al., 2024). Considering the subsequent application of the selected SNP loci for KASP genotyping, the primary screening criterion was set to ensure specificity by requiring each SNP marker to align uniquely to the reference genome within a sequence window of 200 bp upstream and downstream (totaling 401 bp). Furthermore, based on other key criteria, such as the distribution of SNP markers across the genome, the missing base call rate of 0, the MAF greater than 0.2, and the PIC values greater than 0.35, a total of 290 SNP loci were ultimately selected as candidate markers (**Supplementary Table 7**).

**Figure 2:**
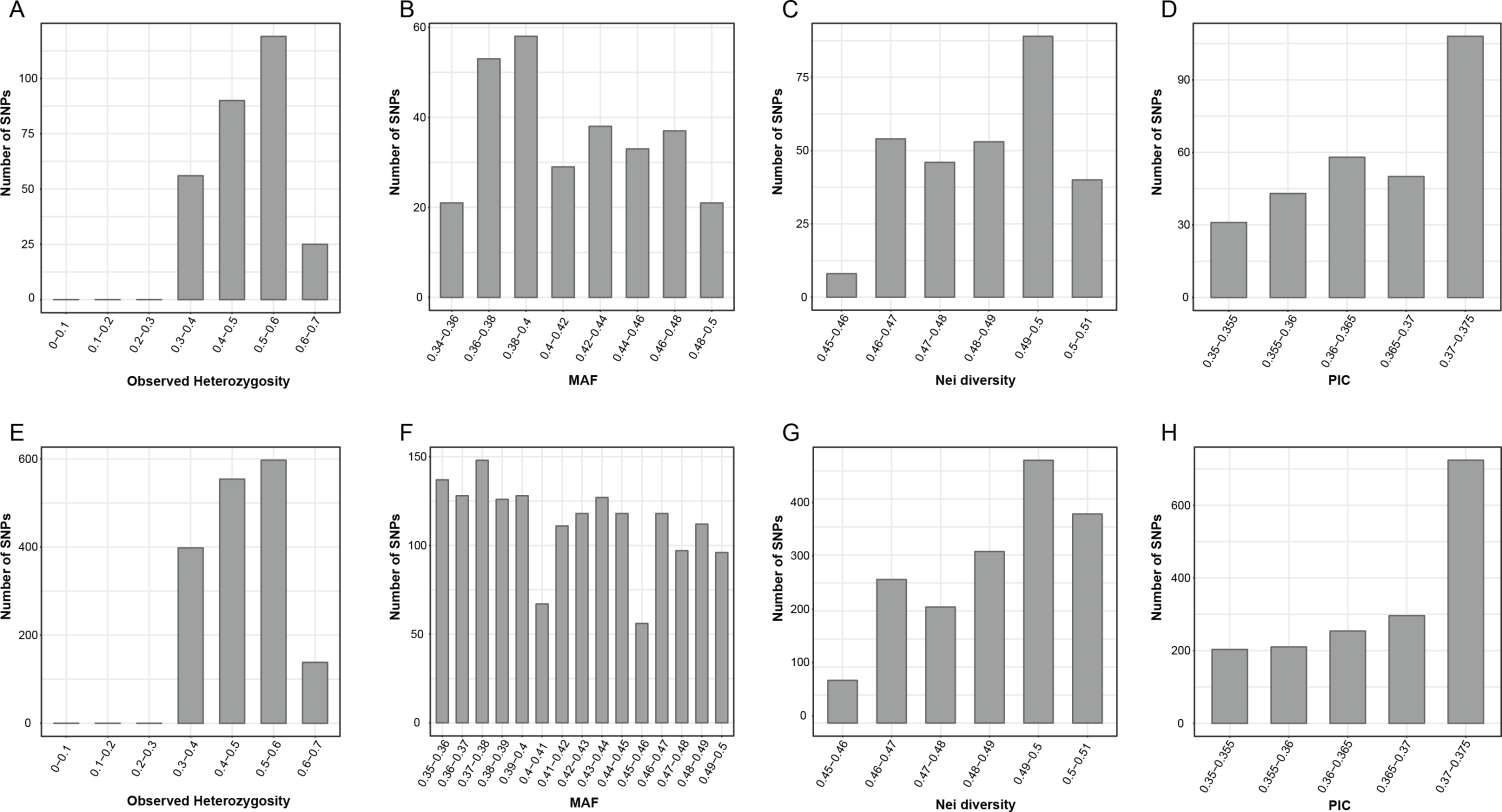
Population genetic analysis of *Elymus sibiricus* accessions based on SNP loci. **(A)** Observed heterozygosity (*H*_o_), **(B)** minor allele frequency (MAF), **(C)** Nei’s diversity index, and **(D)** polymorphism information content (PIC) values of 90 *E. sibiricus* samples based on a set of 290 candidate SNP markers. **(E)** *H*_o_, **(F)** MAF, **(G)** Nei’s diversity index, and **(H)** PIC values of 90 *E. sibiricus* samples based on a set of 338 candidate SNP markers (FP338).

**Figure 3:**
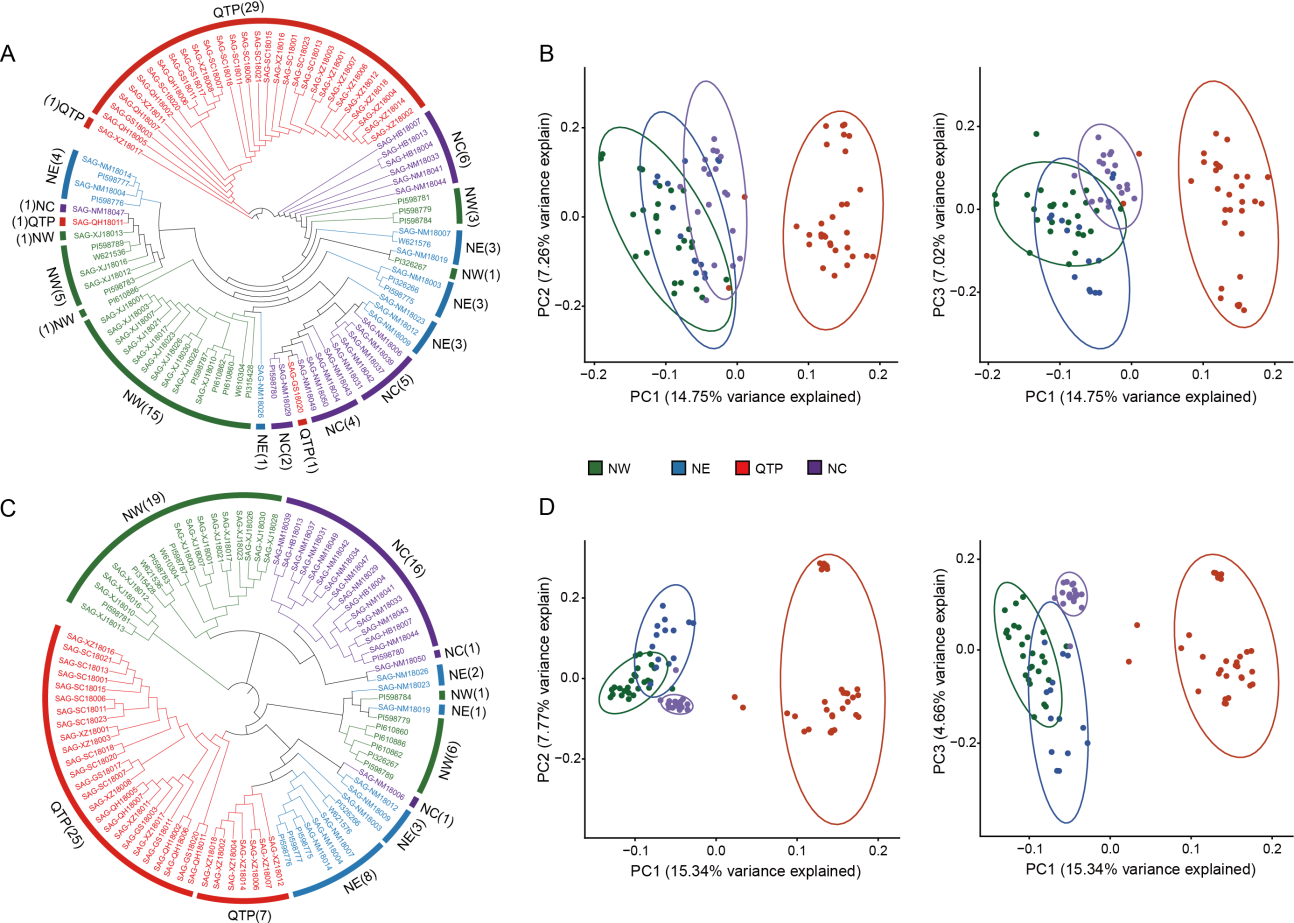
Population structure analysis of 90 *E. sibiricus* samples based on the selected SNP loci. **(A, B)** The phylogenetic tree and principal component analysis (PCA), respectively, of 90 *E. sibiricus* samples based on a set of 290 candidate SNP markers. **(C, D)** The phylogenetic tree and PCA, respectively, of 90 *E. sibiricus* samples based on a set of 338 candidate SNP markers (FP338).

The genetic diversity analysis of the 290 candidate markers revealed that *H*_o_ ranged from 0.333 to 0.633, with an average value of 0.495; the MAF ranged from 0.340 to 0.50, with an average value of 0.415. Additionally, the average values for Nei’s genetic diversity index, the Shannon–Wiener index, and PIC were 0.485, 0.675, and 0.366, respectively (**Figures 2A–D**; **Supplementary Table 8**). These results indicated that the 290 candidate markers exhibited high levels of polymorphism. The accuracy and effectiveness of the candidate markers were assessed through population structure analysis of the 90 wild *E. sibiricus* germplasms using the 290 candidate markers. Through the construction of a phylogenetic tree, it was found that the 290 candidate loci could effectively distinguish *E. sibiricus* germplasms from the QTP population from those of non-QTP populations (including MW, NC, and NE populations) (**Figure 3A**). However, there was a certain degree of error in identifying *E. sibiricus* germplasms from the NW, NC, and NE populations. For example, although 15 *E. sibiricus* germplasms from the NW population clustered together, 11 *E. sibiricus* germplasms from the NW population were scattered among the NE and NC populations. Furthermore, some *E. sibiricus* germplasms from the NC and NE populations were also clustered together. PCA also indicated a clear overlap among the *E. sibiricus* accessions from the NC, NE, and NW populations (**Figure 3B**).

Given that the classification of the NE, NW, and NC populations of *E. sibiricus* germplasms is based on whole-genome SNP data and that there is geographic overlap among these three populations, some materials from the NE and NC populations and NW and NE populations were obtained from locations that are geographically extremely close to each other (**Supplementary Figure 2**). Therefore, we hypothesize that it is challenging to completely and accurately distinguish accessions from the NE, NC, and NW populations from each other using a simple set of SNP loci combinations alone. To test this hypothesis, we abandoned the specificity criteria for SNP locus selection and constructed a set of candidate markers comprising 338 SNPs (FP338) with higher genetic diversity (**Figures 2E–H**; **Supplementary Tables 8**, **9**). Using the FP338 loci, a phylogenetic tree and PCA were conducted for all 90 *E. sibiricus* germplasm accessions (**Figures 3C, D**). Using this subset of SNPs, it was still not possible to completely distinguish the materials from the NE, NC, and NW populations, with seven accessions from the NW population, six from the NE population, and two from the NC population still failing to be accurately classified. Notably, in the classification results using the FP338 candidate markers, seven germplasms from the QTP population (SAG-XZ18018, SAG-XZ18002, SAG-XZ18004, SAG-XZ18014, SAG-XZ18006, SAG-XZ18007, and SAG-XZ18012) were clustered together. Additionally, the genetic distances among these seven germplasms were also closer in the clustering results based on the 290 SNP loci, which was consistent with the results of the delineation of QTP populations accessions based on genome-wide SNPs, indicating that these candidate loci effectively reflect the genetic similarities among these germplasms.

#### 3.2.2 Screening of core SNP markers and construction of DNA fingerprints

**Figure 4:**
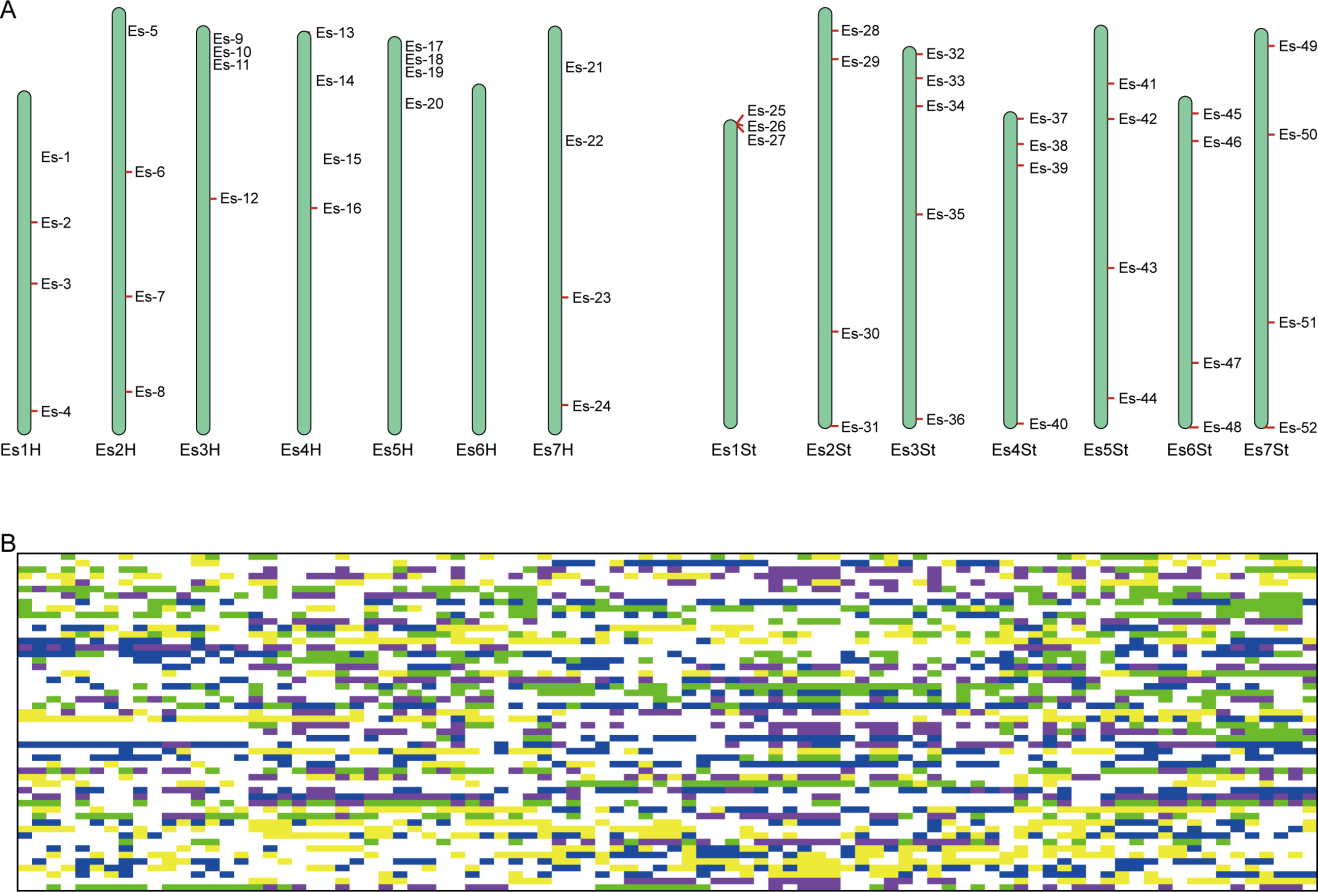
Fingerprint analysis of 90 *Elymus sibiricus* samples. **(A)** The distribution of 52 core SNP markers on 14 *E. sibiricus* chromosomes. **(B)** DNA fingerprint composed of 52 core SNP markers. Each row corresponds to a SNP marker, and each column corresponds to a *E. sibiricus* sample. The genotypes C/C, A/A, T/T, and G/G are represented in yellow, green, blue, and purple, respectively. Missing sites are represented in gray, and heterozygous sites are shown in white.

**Figure 5:**
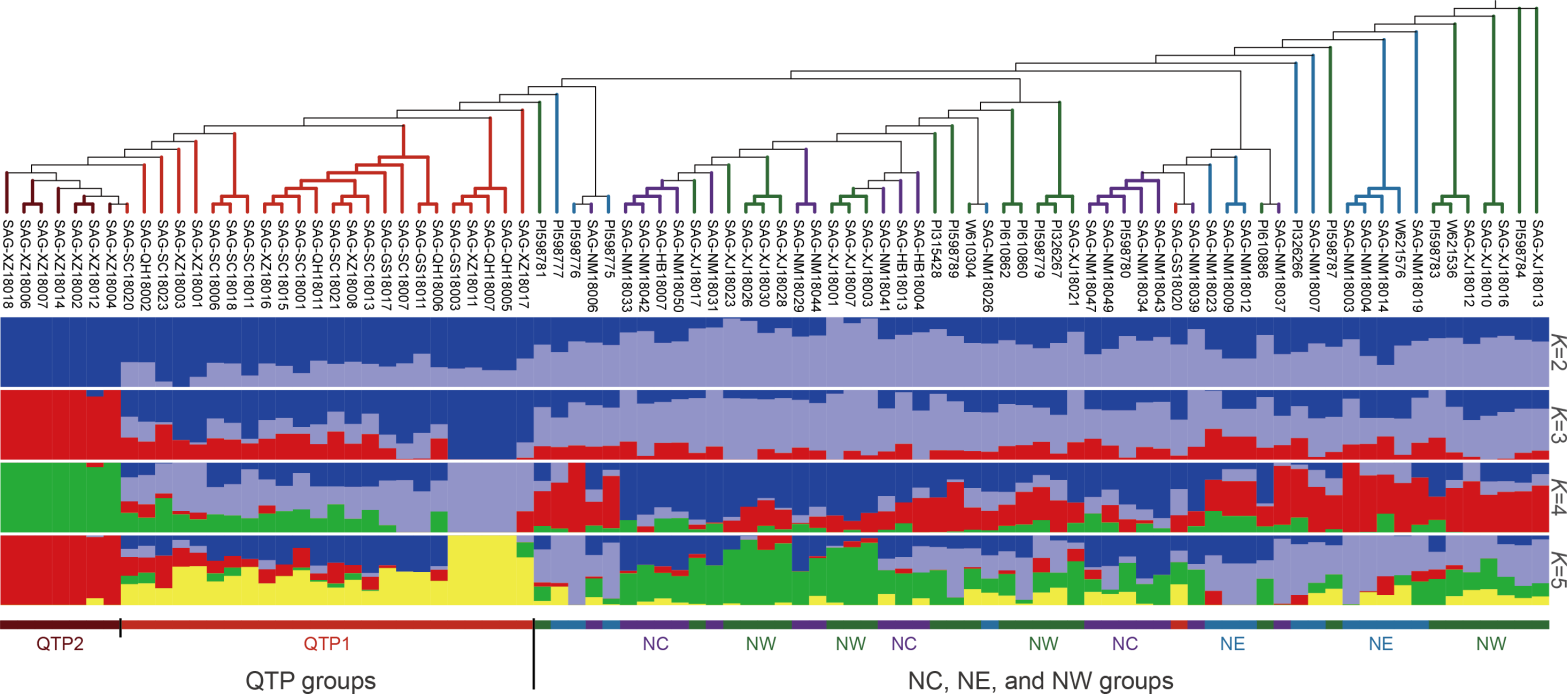
Population structure analysis of 90 *Elymus sibiricus* samples based on the 52 core SNP markers. From top to bottom, the figure shows a phylogenetic tree of 90 *E. sibiricus* samples based on 52 core SNP markers, the sample names, and the population structure of 90 *E. sibiricus* germplasm resources for different numbers of subpopulations *K*.

Despite the limitations of the 290 candidate SNPs for use in identifying the population location of some *E. sibiricus* germplasm accessions from NE, NC, and NW populations, they could still be used to accurately predict the source of germplasm from the QTP population, as well as distinguish differences among individual germplasm accessions. In order to rapidly and economically distinguish *E. sibiricus* germplasm sources, 52 core SNPs were ultimately selected after screening for loci with high PIC and MAF values from a pool of 290 candidate markers, and ensuring that these SNP loci were evenly distributed across the 14 chromosomes of *E*. *sibiricus*, with the genotypes C/C, A/A, T/T, and G/G represented by yellow, green, blue, and purple, respectively; additionally, missing sites are represented in gray, and heterozygous sites are shown in white (**Figure 4A**; **Supplementary Data 2**). Using this set of core SNP loci, pairwise comparisons were conducted for the 90 *E. sibiricus* samples, and the results successfully distinguished each *E. sibiricus* germplasm accession (**Figure 4B**). Furthermore, through the analysis of the population structure of the 90 *E. sibiricus* samples, it was possible to accurately differentiate germplasms from the QTP population from those obtained from non-QTP populations, which indicated that the 52 core loci indeed effectively represent the 290 candidate SNPs (**Figure 5**). Genotyping data for the 52 core SNPs of the 90 *E. sibiricus* germplasms were encoded using the online software Caoliaoerweima (http://cli.im/), and 2D barcode fingerprints were generated for each germplasm (**Supplementary Data 3**).

#### 3.2.3 Design of KASP primers and identification of *E. sibiricus* germplasm resources

The accuracy of analysis based on the 52 core SNP loci was further evaluated via PCR and Sanger sequencing for nine samples (SAG-NM18033, SAG-HB18004, SAG-XJ18028, SAG-XJ18013, SAG-NM18050, SAG-SC18007, SAG-GS18003, SAG-XZ18007, and PI598784) that were randomly selected from among the 90 samples. We found that the verification results for some SNP loci deviated from those obtained by next-generation sequencing, leading to reduced accuracy and reduced specificity of the loci. Ultimately, only 31 out of the 52 core SNPs could be successfully converted into KASP markers (**Supplementary Table 10**).

To evaluate the effectiveness of these 31 KASP markers in classifying *E. sibiricus* germplasms, 16 samples were randomly selected from among the 90 germplasm accessions and subjected to KASP analysis along with 44 other *E. sibiricus* germplasm accessions from the NC, NW, NE, and QTP populations (**Supplementary Table 2**). We observed that 27 KASP markers demonstrated a high discriminatory power, which were able to achieve distinct genotyping results in 60 samples (**Supplementary Data 4**). However, there were four KASP markers (KASP-SNP14, KASP-SNP20, KASP-SNP28, KASP-SNP18) with relatively poor typing results. In some samples, the homozygous or heterozygous status of the corresponding SNP sites could not be accurately identified, which might be related to the specificity of these loci in different samples. A genetic distance matrix and a phylogenetic tree were constructed based on the genotyping results (**Supplementary Table 11**). Thus, accessions from the QTP population were separated by smaller genetic distances and were clearly clustered together in the phylogenetic tree (**Supplementary Figure 3**). However, three samples from outside the QTP population (NC-test12, NC-test9, and NE-test6) were mixed into this cluster, such that the genetic distance between NC-test9 and QTP-test4 was 0.0556, that between NE-test6 and QTP-test5 was 0.1429, and that between NC-test12 and QTP-test8 was 0. This unexpected clustering may be related to the failure of some SNP loci to serve as functional KASP markers. Furthermore, there remained a lack of clarity in the population source identification of germplasms from the NW, NC, and NE populations. For example, two samples from the NC population (NC-test4 and SAG-NM18037) and four samples from the NE population (NE-test9, NE-test10, NE-test5, and PI598775) clustered with most of the samples from the NW population. Additionally, three samples from the NE population (NE-test11, NE-test8, and NE-test1) were also clustered with eight germplasms from the NC population. These results again highlight the limitations of using a small number of SNP locus combinations to differentiate *E. sibiricus* germplasm accessions with similar genetic backgrounds.

## 4 Discussion

As a widely distributed species across the Eurasian continent, the wild germplasm resources of *E. sibiricus* are extremely rich and abundant (Ma et al., 2012). Influenced by climatic factors in different habitats, the genetic variation among *E. sibiricus* accessions exhibits distinct regional characteristics (Li et al., 2023e); for instance, the phenology of heading and the presence of small spines at the base of the stems and leaf sheaths in wild *E. sibiricus* are significantly associated with high altitude (Li et al., 2023a). The phenotype characterized by the appearance of downy hair on the basal leaf sheaths during the seedling stage is found to have an extremely significant positive correlation with both longitude and latitude, while it demonstrates an extremely significant negative correlation with altitude, annual mean temperature, and annual average rainfall (Li et al., 2023d). Genetic variation in these traits, which manifest clear regional characteristics, may represent adaptations of *E. sibiricus* germplasm under different environmental conditions, not only enriching the genetic diversity of *E. sibiricus* but also promoting population differentiation and providing an important genetic resource foundation for the development and breeding of new *E. sibiricus* varieties with superior traits (Zhang et al., 2022; Xiong et al., 2024a). Furthermore, determining how to scientifically identify, preserve, and protect these wild *E. sibiricus* germplasm resources is a prerequisite for their effective utilization. The genetic diversity of multiple important crops, including sorghum (Cuevas et al., 2017), sweet potato (Su et al., 2017), pumpkin (Lee et al., 2020), cowpea (Ketema et al., 2020), radish (Xing et al., 2024), and wheat (Soleimani et al., 2020), has been preserved as much as possible through the construction of a core germplasm collection based on SNP markers. However, in the construction of a core germplasm collection for *E. sibiricus*, the focus has primarily remained on phenotypic traits and the use of SSRs as markers. Yan et al. (2017) used SSR markers to identify 47 accessions as the core germplasm collection from among 148 samples of *E. sibiricus*. Zeng et al. (2022) identified five accessions as the core germplasm collection for breeding by evaluating nine agronomic traits of 76 *E. sibiricus* germplasm accessions. The observation of plant phenotypic traits is one of the more traditional and intuitive methods of core germplasm identification. However, most of the phenotypic traits in plants are quantitative traits affected by multiple minor-effect genes and are therefore often susceptible to environmental influences (Wang et al., 2021a; Mackay and Anholt, 2024). Based on literature reports and field observations, we found that even the same *E. sibiricus* germplasm accession could exhibit significant phenotypic differences under different environmental conditions (Zhang, 2020; Jia, 2021). Additionally, there is substantial variation in the phenotypes of *E. sibiricus* across different growth years. Specifically, the agronomic traits of *E. sibiricus* are generally optimal during the second and third years of growth after plants are established, showing considerable differences from those in the first year, indicating the need for a longer period for phenotypic characterization of *E. sibiricus* germplasm accessions (Zhou et al., 2000; Chen, 2013; Qi et al., 2023). These factors present a substantial hindrance in the construction of a core germplasm bank for *E. sibiricus* using phenotypic traits. Additionally, although SSR markers are widely used in the construction of plant core germplasm collections, SNP markers offer several advantages: they are more abundant, have higher density, exhibit greater levels of polymorphism, and are more stable (Rafalski, 2002; Mammadov et al., 2012). Moreover, the identification and statistical analysis of SNP markers are even easier and more convenient through the utilization of the high-throughput automatic detection capabilities of the KASP genotyping platform (Smith and Maughan, 2015; Dipta et al., 2024). Therefore, we identified 36 *E. sibiricus* accessions as the core germplasm collection based on SNP markers, which can accurately represent the genetic diversity of the original 90 *E. sibiricus* germplasm accessions. This study represents the initial exploration of constructing a core germplasm resource for *E. sibiricus* based on a large number of SNP markers, providing a valuable reference for future collection and preservation of *E. sibiricus* germplasm resources. Concurrently, this core collection not only effectively protect the genetic diversity of *E. sibiricus* germplasm and prevent the loss of resources, but also serve as the basic material for breeding, providing important genetic resources for the breeding of new varieties.

Currently, the identification of wild germplasm resources of *E. sibiricus* mainly focuses on distinguishing *E. sibiricus* germplasm from congeneric *E*. *nutans* germplasm. Owing to the rich phenotypic variation in the wild germplasm of these two forage grasses, some of the wild germplasm of the two species are morphologically very similar, and the regions of their geographic distributions overlap. Li et al. (2023d) identified 990 *E. sibiricus* accessions and 246 *E. nutans* accessions from 1,723 wild *Elymus* germplasm resources by combining phenotypic trait analysis with flow cytometry. However, the population source identification of wild *E. sibiricus* germplasms has not been reported. The construction of fingerprints based on SNPs and the combination of KASP for typing identification can be utilized to accurately and efficiently differentiate among various germplasm sources within a species. This approach has been widely applied in the identification of varieties and population structure analysis in many species (Yang et al., 2019; Wang et al., 2021b, 2022; Yang et al., 2022). Therefore, in this study, based on the SNP markers obtained from the whole-genome sequencing data of 90 wild *E. sibiricus* accessions from the QTP, NC, NE and NW populations, we initially explored the feasibility of identifying the population origins of wild *E. sibiricus* germplasm accessions using SNP fingerprints combined with KASP genotyping. Unlike diploid species such as maize and cigar tobacco that have already established SNP fingerprints (Tian et al., 2021; Wang et al., 2021a), *E. sibiricus*, as an allotetraploid species, presents challenges in precise SNP genotyping due to the presence of homologous chromosomes between sub-genomes and a large number of repetitive sequences on the chromosomes, which result in many markers showing multiple copy phenomena (Yan et al., 2024). Therefore, we prioritized the specificity of SNPs as the primary criterion for selecting candidate SNP sites for fingerprint construction, in order to screen SNP sites with single-copy characteristics, thus simplifying the complex polyploid genotyping to a diploid-like genotyping, and facilitating KASP detection. However, similar to findings in the construction of SNP fingerprints for allotetraploid upland cotton (*Gossypium hirsutum* L.), this screening criterion limits the number of available SNPs and genetic diversity (Sun, 2017). In this study, the selected core SNP markers were able to accurately discriminate germplasm from QTP and non-QTP populations, but there were obvious errors in discriminating germplasm from NC, NE and NW populations. Some of the accessions from these three populations were clustered together based on the genotyping results, which indicated that the selected SNP markers lacked sufficient discriminatory power for differentiating the germplasm from these three populations. We speculate that this result may be related to the similar genetic backgrounds of individuals from these three populations. The materials from geographically closer populations may exhibit more genetic homogeneity owing to both environmental similarities as well as gene flow between populations. Consequently, it is challenging to accurately identify their population source using only a few SNP loci. For the QTP population of *E. sibiricus*, there was not only a large geographic distance from the NE, NC, and NW populations, but also genetic differences likely shaped by the unique environmental conditions of the QTP, such as its high altitude, which may lead to significant genetic differentiation from the non-QTP populations. Accordingly, even a small number of SNP loci were sufficient to distinguish germplasms from the QTP population from those from other populations. The similar findings have been reported in studies on tobacco (Wang et al., 2021a) and honeysuckle (Li et al., 2023b) fingerprint maps. Additionally, we also infer that a more comprehensive collection and sequencing analysis of *E. sibiricus* germplasm from the regions corresponding to these three populations would obtain richer SNP information. This will not only facilitate more detailed population differentiation of accessions from these regions but also help construct more discriminative SNP fingerprints, thereby playing a crucial role in identifying the population origins of *E. sibiricus* accessions. It is noteworthy that Yan et al. (2024), based on SNP datasets obtained from whole-genome sequencing, found that even *E. sibiricus* germplasm with close geographical origins were classified into different populations. Therefore, compared to the SCoT molecular markers used in previous study to identify the geographical origin of *E. sibiricus* (Xie et al., 2015), the DNA fingerprints constructed based on SNP markers in this study can identify the population origin of *E. sibiricus* germplasm, which is more beneficial for analyzing the population structure of *E. sibiricus* germplasm. Furthermore, studies have indicated that the *E. sibiricus* germplasm from the QTP population possesses higher genetic diversity, and it is inferred that the Qinghai-Tibet Plateau is very likely the center of origin for *E. sibiricus* (Xiong et al., 2024b; Yan et al., 2024). Consequently, it is of significant importance for the conservation, rational utilization and molecular breeding of *E. sibiricus* germplasm resources to rapidly discern whether the *E. sibiricus* germplasm originates from the QTP population (QTP) through fingerprints.

## 5 Conclusion

In this study, we successfully constructed a core collection comprising 36 *E. sibiricus* samples by integrating published sequencing data from 90 wild *E. sibiricus* accessions. Additionally, 290 candidate SNP markers and 52 core SNP markers were identified for the development of *E. sibiricus* DNA fingerprints, according to a series of strict screening criteria and evaluation methods. Subsequently, utilizing KASP technology, we genotyped 60 wild *E. sibiricus* accessions using these core SNP markers. The core SNP makers were able to accurately identify the germplasms from the QTP population, but there were some errors in the population origin identification of *E. sibiricus* germplasm from the NC, NE, and NW populations. We speculate that this result is owing to the relatively similar genetic backgrounds of *E*. *sibiricus* from these three populations. Therefore, we advocate for a more comprehensive collection and sequencing analysis of *E. sibiricus* germplasm resources from the regions corresponding to these three populations, which would enrich the SNP database and facilitate more precise analyses of population structure and gene flow in these areas, as well as the selection of more effective DNA marker combinations. In summary, our study preliminarily validated the feasibility of constructing a core germplasm set and DNA fingerprint for *E. sibiricus* based on SNPs. This work not only underscores the reliability and precision of SNP markers but also lays a crucial foundation for future efforts in the collection, conservation, and utilization of *E. sibiricus* germplasm resources.

## Data Availability

The original contributions presented in the study are included in the article/**Supplementary Material**. Further inquiries can be directed to the corresponding author.

## Supplementary material

Supplementary Figure 1 Distribution of minor allele frequency (MAF) values in the core collection and the original full germplasm collection. Intervals of MAF values corresponding to SNPs are shown along the *x*-axis; the *y*-axis represents the frequency of all SNPs in each interval.

Supplementary Figure 2 Geographic distribution of 90 Elymus *sibiricus* samples, both at a broad scale and locally.

Supplementary Figure 3 Clustering analysis dendrogram of 60 wild *Elymus sibiricus* germplasm accessions based on genotyping results for 31 KASP markers. The bands next to the dendrogram indicate the population origin of the samples, where red, purple, blue, and green represent *E. sibiricus* samples from the QTP, NC, NE, and NW regions, respectively.
