# Supplementary figures and images for: An initial exploration of core collection construction and DNA fingerprinting in Elymus sibiricus L. using SNP markers
Source: Front Plant Sci. 2025 Feb 7;16:1534085. doi: 10.3389/fpls.2025.1534085 (PMC11844813; doi:10.3389/fpls.2025.1534085)

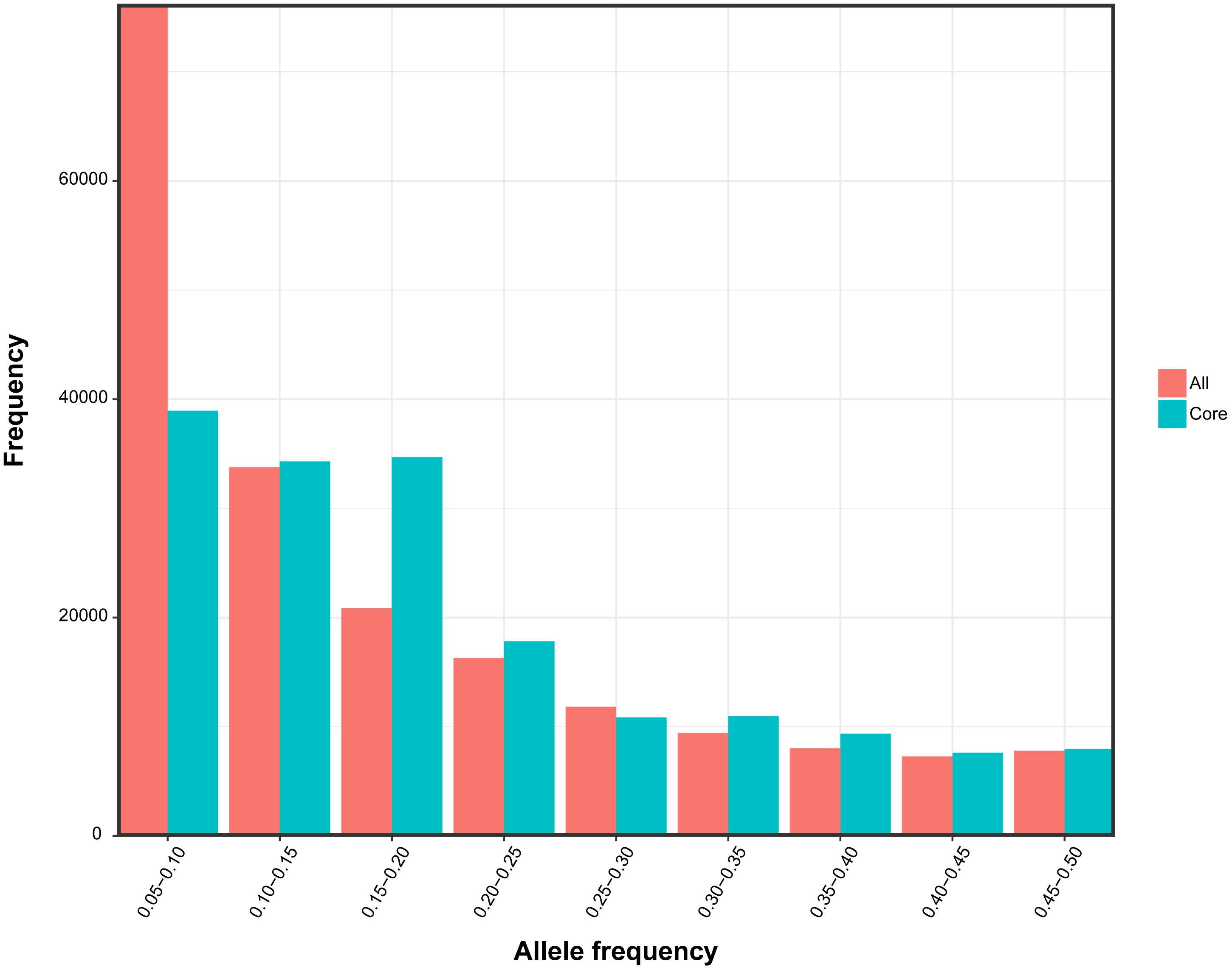

Supplement: Supplementary Figure 1 — Distribution of minor allele frequency (MAF) values in the core collection and the original full germplasm collection. Intervals of MAF values corresponding to SNPs are shown along the x-axis; the y-axis represents the frequency of all SNPs in each interval. [file Image1.tif]

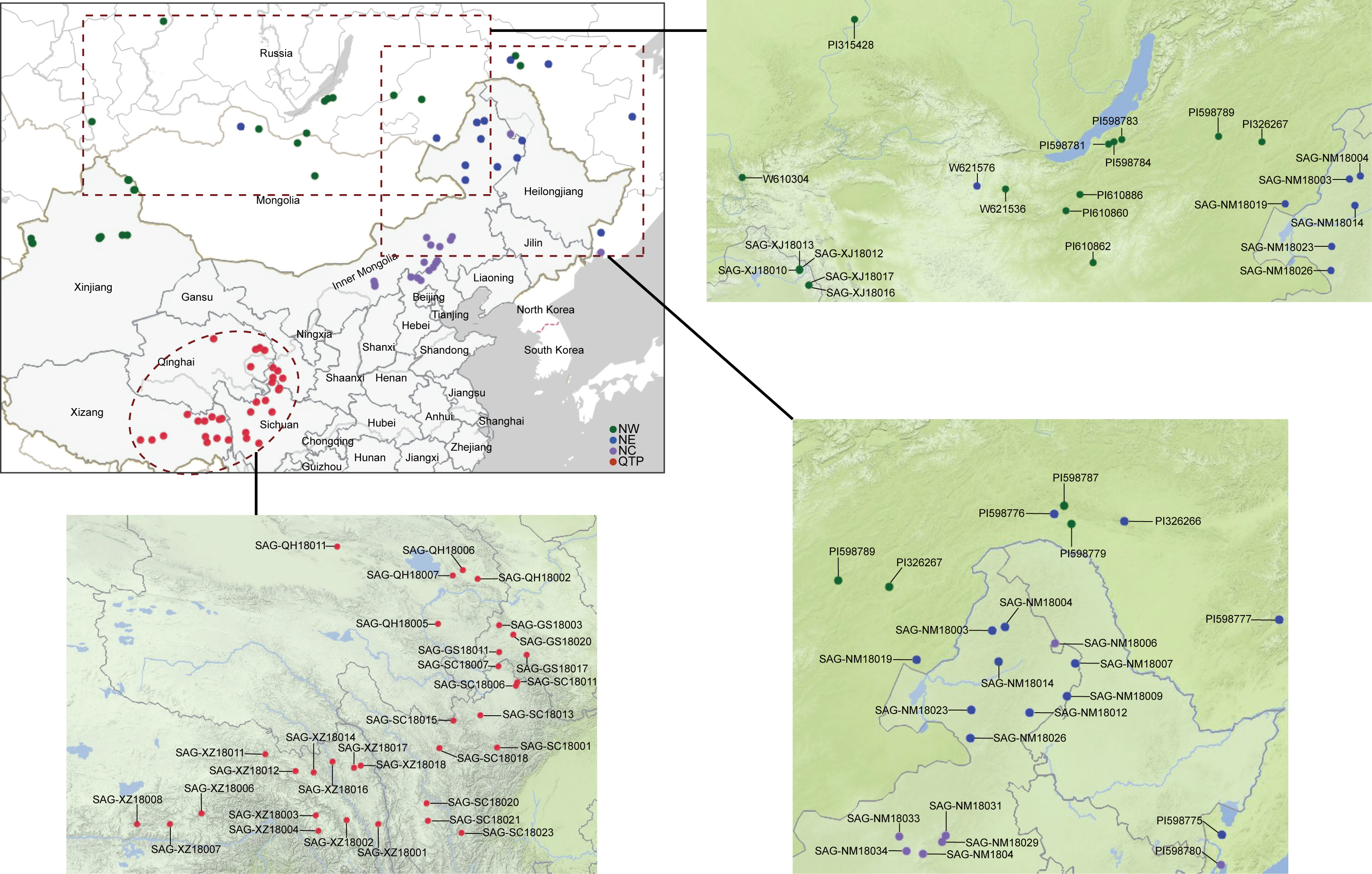

Supplement: Supplementary Figure 2 — Geographic distribution of 90 Elymus sibiricus samples, both at a broad scale and locally. [file Image2.tif]

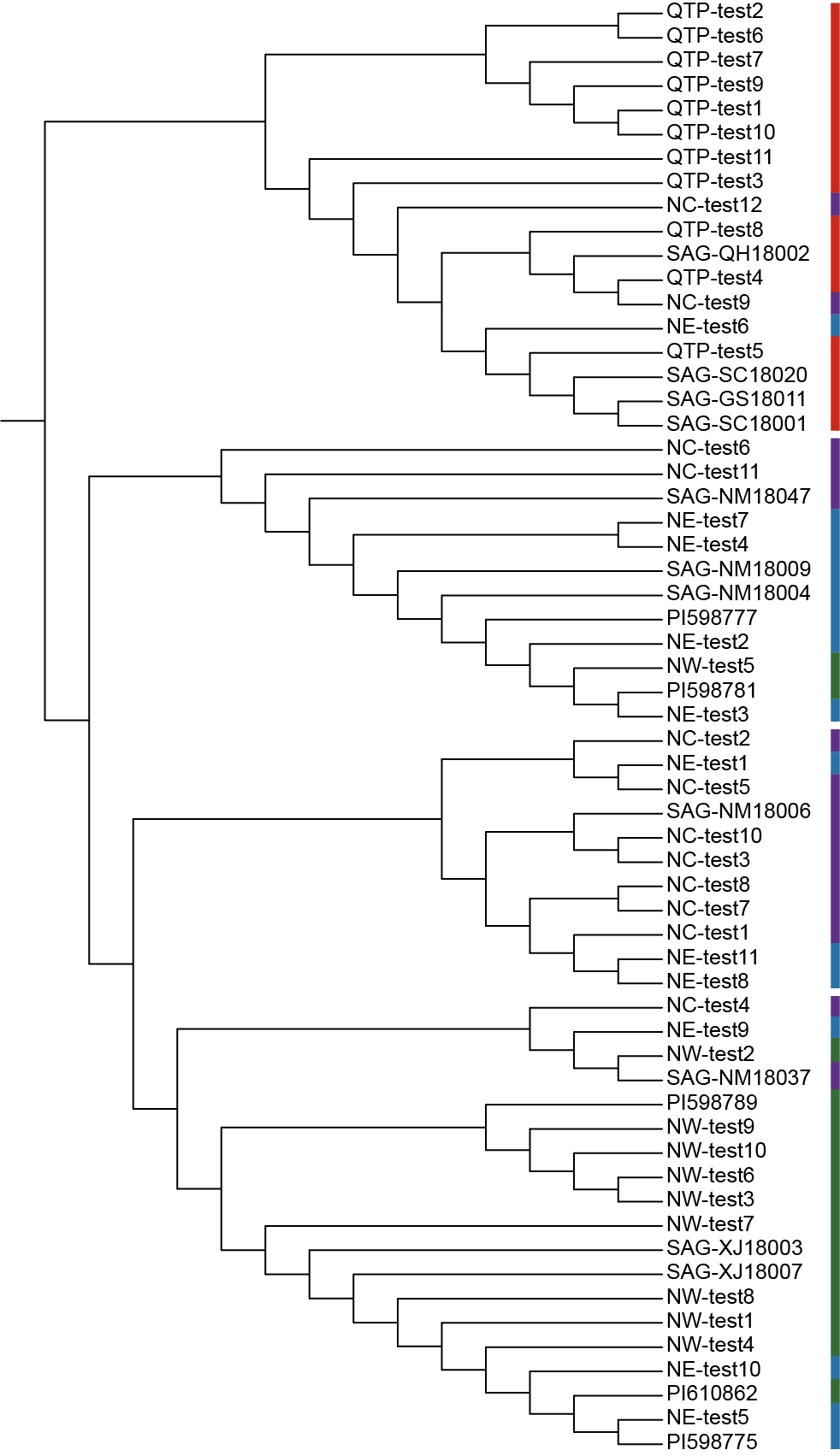

Supplement: Supplementary Figure 3 — Clustering analysis dendrogram of 60 wild Elymus sibiricus germplasm accessions based on genotyping results for 31 KASP markers. The bands next to the dendrogram indicate the population origin of the samples, where red, purple, blue, and green represent E. sibiricus samples from the QTP, NC, NE, and NW regions, respectively. [file Image3.tif]
