# Supplementary material for: An initial exploration of core collection construction and DNA fingerprinting in Elymus sibiricus L. using SNP markers
Source: Front Plant Sci. 2025 Feb 7;16:1534085. doi: 10.3389/fpls.2025.1534085 (PMC11844813; doi:10.3389/fpls.2025.1534085)
Supplement: Supplementary file 6 [file DataSheet3.pdf]

W610304	SAG-XJ18030	SAG-XJ18028	SAG-XJ18026
SAG-XJ18023	SAG-XJ18021	SAG-XJ18017	SAG-XJ18016
SAG-XJ18013	SAG-XJ18012	SAG-XJ18010	SAG-XJ18007
SAG-XJ18003	SAG-XJ18001	PI326266	PI598779
PI598776	PI598787	PI598777	PI598780

PI598775	SAG-QH18011	SAG-QH18007	SAG-QH18006
SAG-QH18005	SAG-QH18002	SAG-XZ18018	SAG-XZ18017
SAG-XZ18016	SAG-XZ18014	SAG-XZ18012	SAG-XZ18011
SAG-XZ18008	SAG-XZ18007	SAG-XZ18006	SAG-XZ18004
SAG-XZ18003	SAG-XZ18002	SAG-XZ18001	SAG-SC18023

SAG-SC18021	SAG-SC18020	SAG-SC18018	SAG-SC18015
SAG-SC18013	SAG-SC18011	SAG-SC18007	SAG-SC18006
SAG-SC18001	SAG-GS18020	SAG-GS18017	SAG-GS18011
SAG-GS18003	PI326267	PI315428	PI598783
PI598781	PI598784	PI598789	W621576

W621536	PI610860	PI610862	PI610886
SAG-NM18026	SAG-NM18023	SAG-NM18019	SAG-NM18014
SAG-NM18012	SAG-NM18009	SAG-NM18007	SAG-NM18006
SAG-NM18004	SAG-NM18003	SAG-HB18013	SAG-HB18007
SAG-HB18004	SAG-NM18050	SAG-NM18049	SAG-NM18047

SAG-NM18044 | SAG-NM18043 | SAG-NM18042 | SAG-NM18041

SAG-NM18039 | SAG-NM18037 | SAG-NM18034 | SAG-NM18033

SAG-NM18031 | SAG-NM18029
