# Supplementary material for: An initial exploration of core collection construction and DNA fingerprinting in Elymus sibiricus L. using SNP markers
Source: Front Plant Sci. 2025 Feb 7;16:1534085. doi: 10.3389/fpls.2025.1534085 (PMC11844813; doi:10.3389/fpls.2025.1534085)
Supplement: Supplementary file 7 [file DataSheet4.pdf]

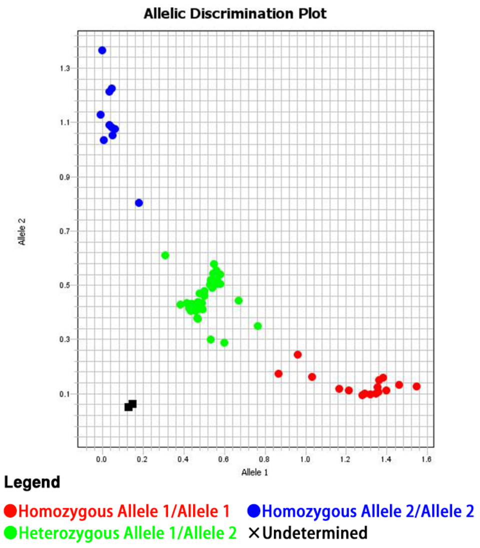

KASP-SNP1

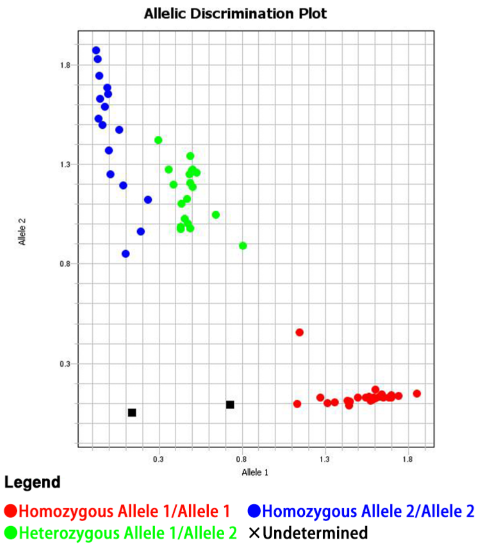

KASP-SNP2

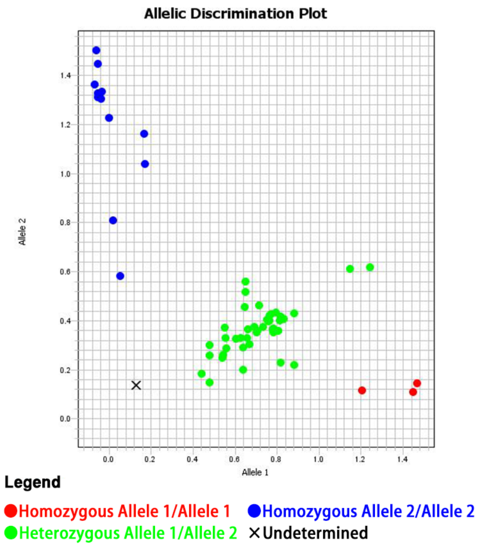

KASP-SNP5

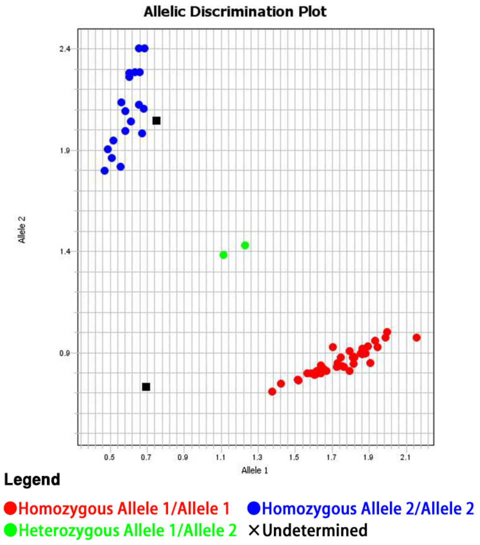

KASP-SNP6

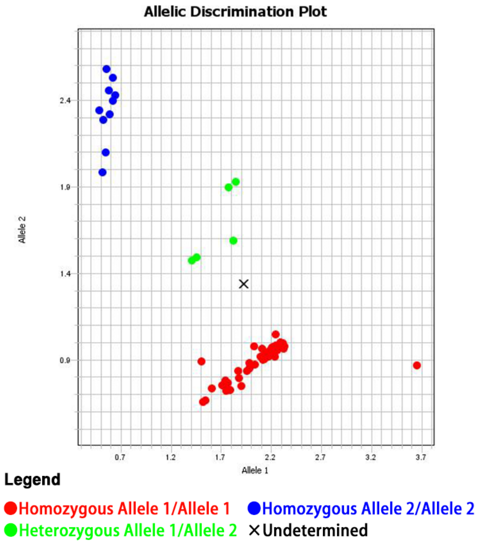

KASP-SNP7

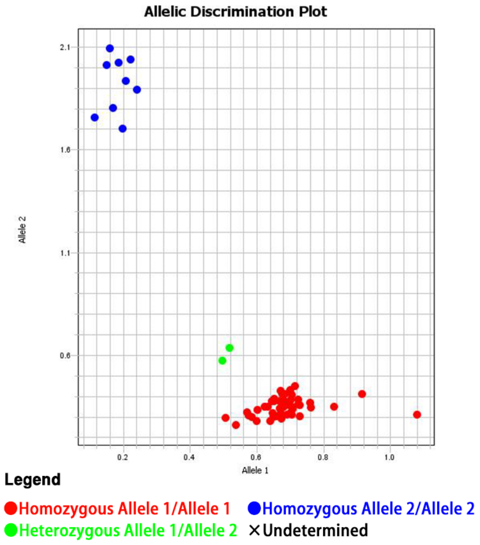

KASP-SNP8

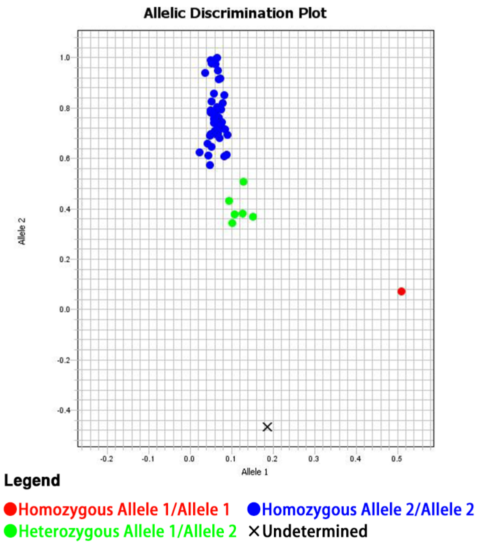

KASP-SNP10

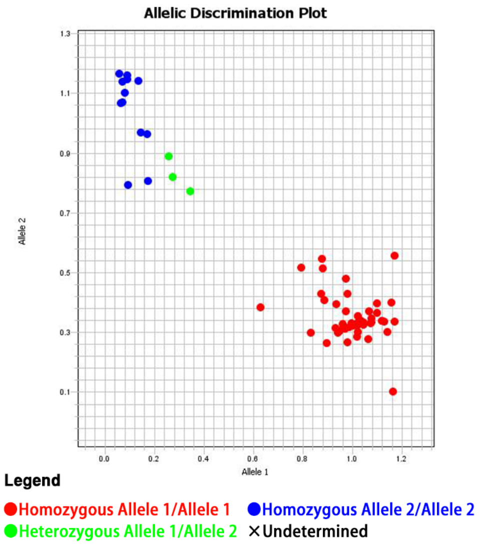

KASP-SNP11

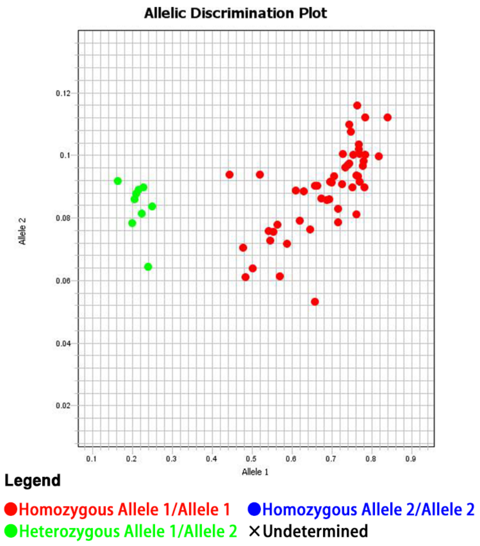

KASP-SNP15

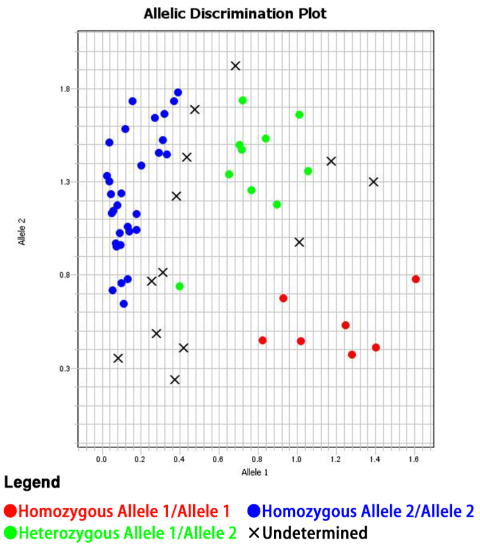

KASP-SNP16

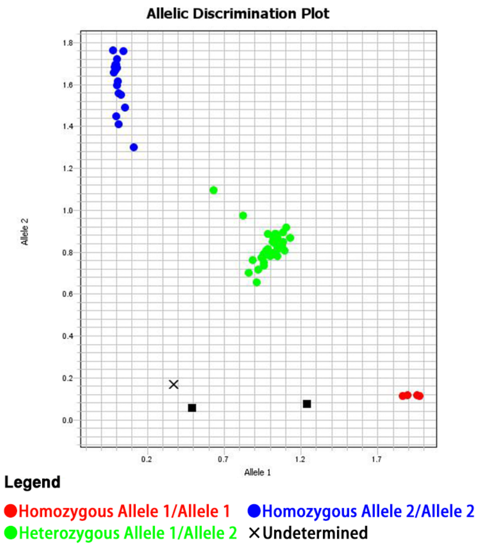

KASP-SNP17

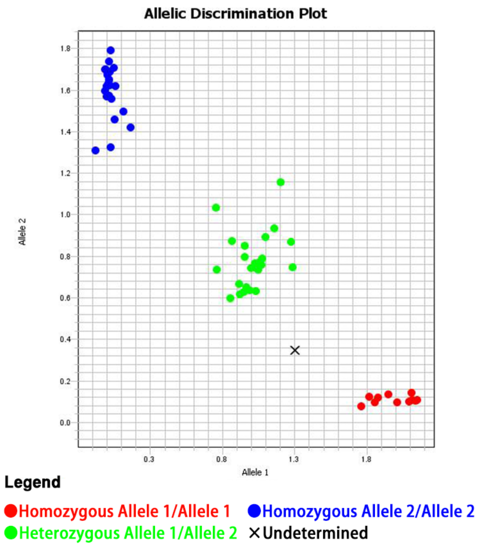

KASP-SNP26

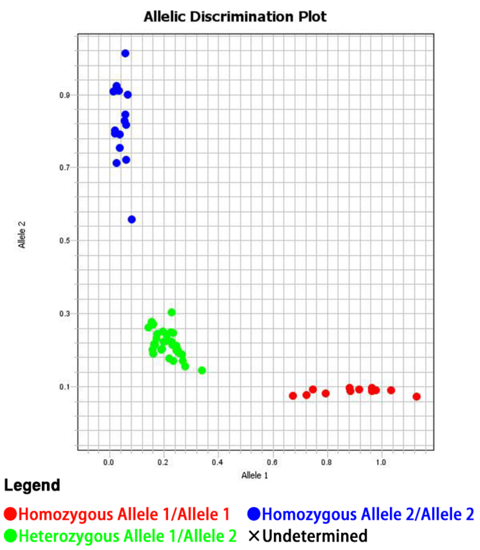

KASP-SNP27

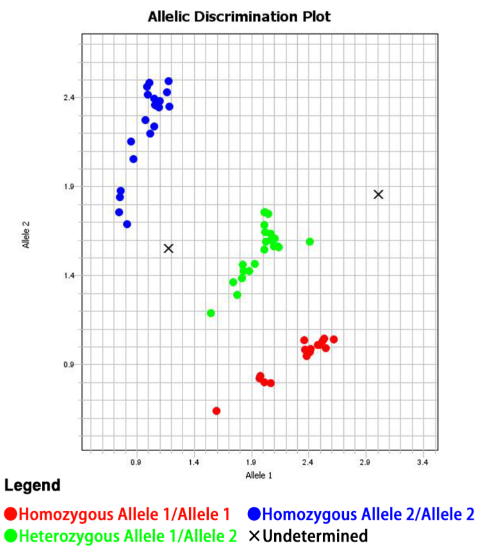

KASP-SNP29

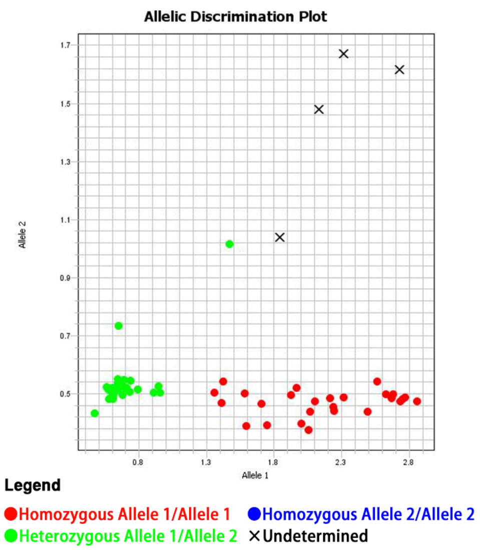

KASP-SNP3

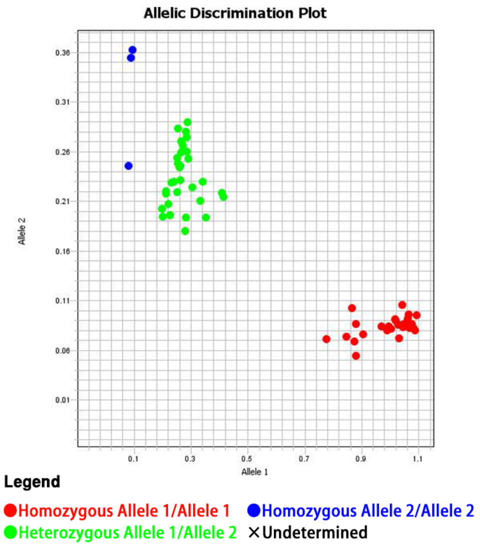

KASP-SNP4

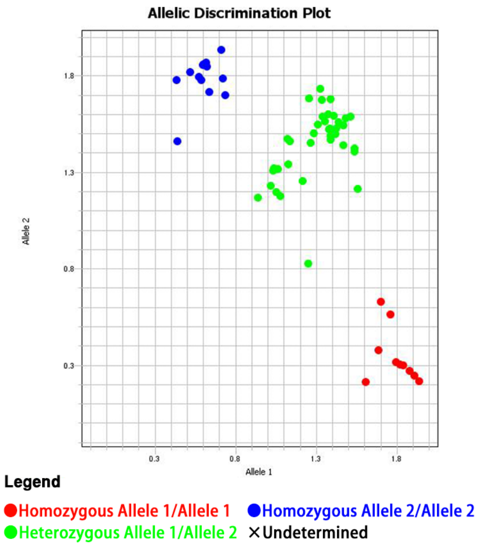

KASP-SNP9

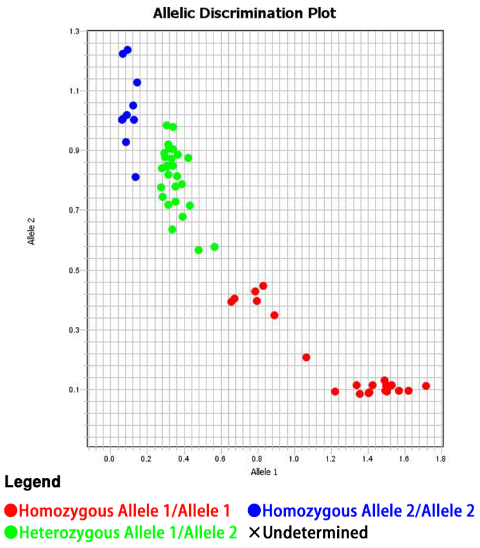

KASP-SNP12

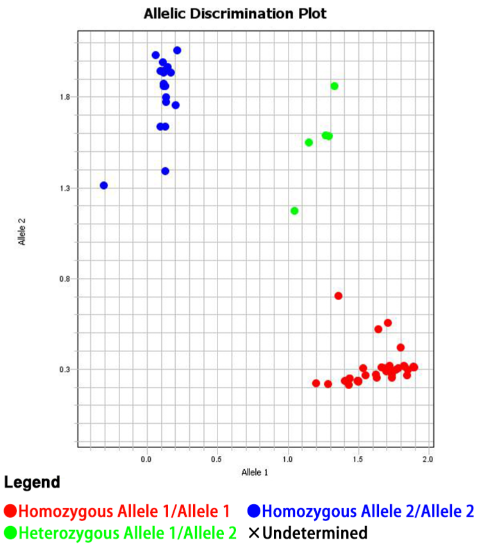

KASP-SNP13

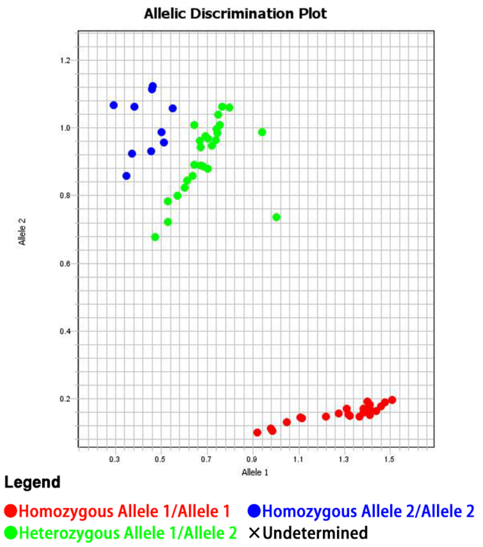

KASP-SNP19

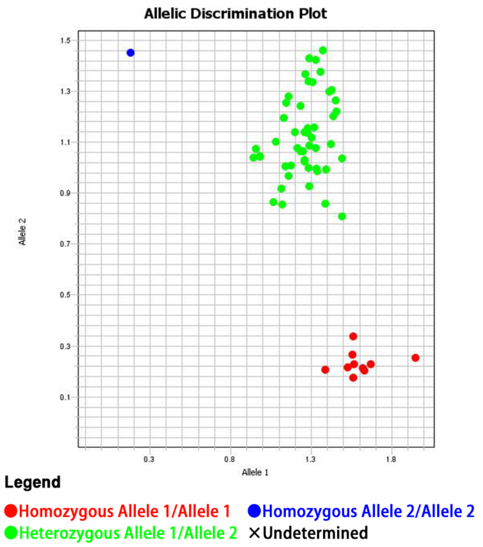

KASP-SNP21

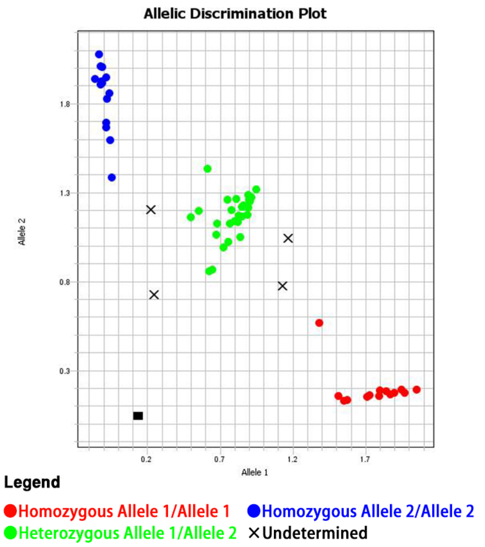

KASP-SNP22

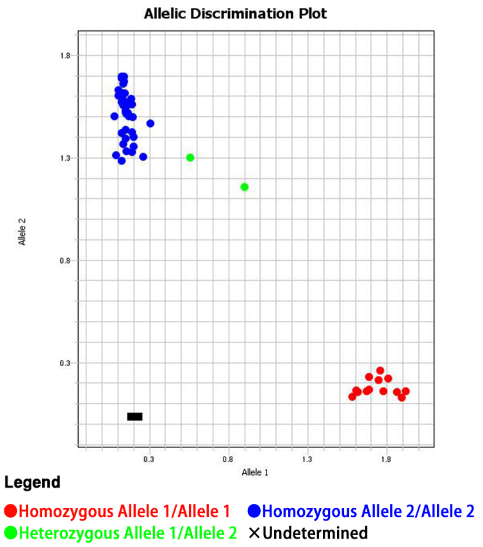

KASP-SNP23

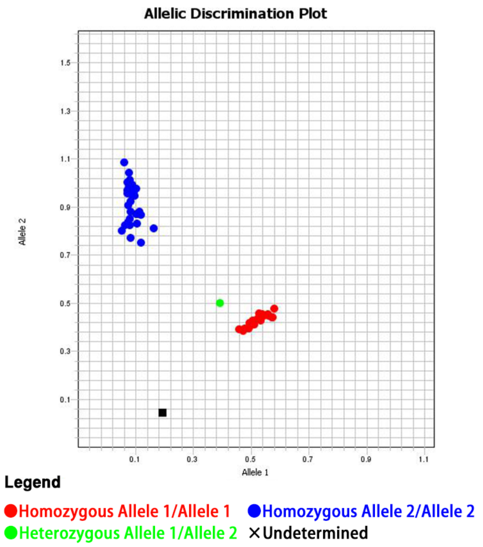

KASP-SNP24

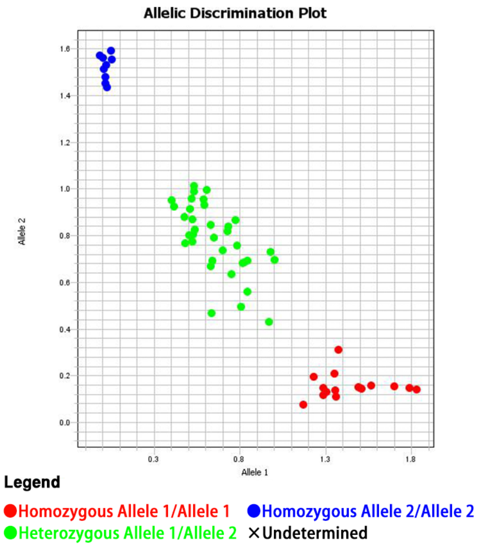

KASP-SNP25

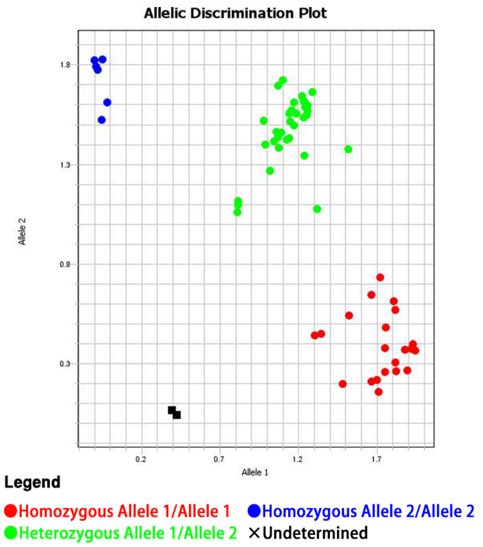

KASP-SNP30

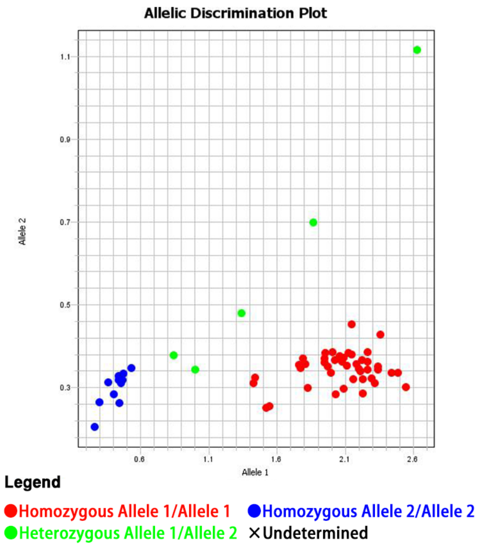

KASP-SNP31

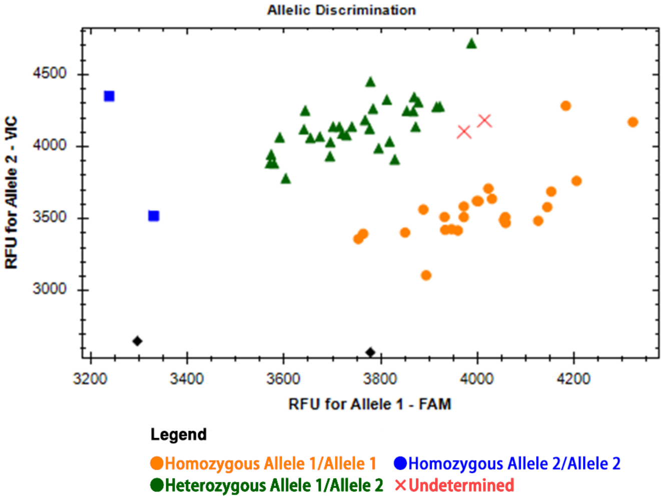

KASP-SNP14

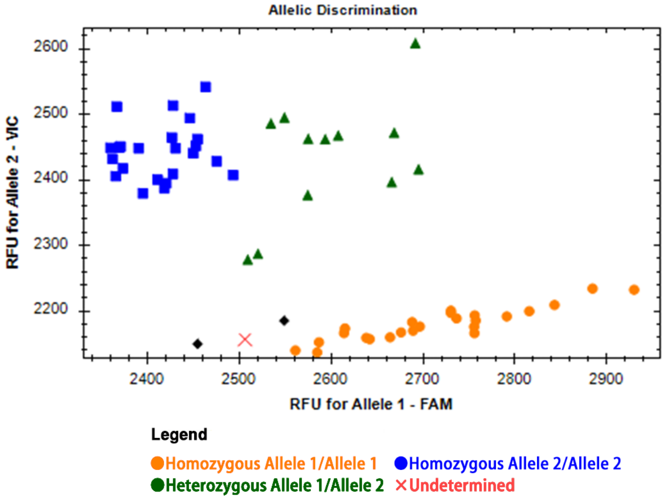

KASP-SNP20

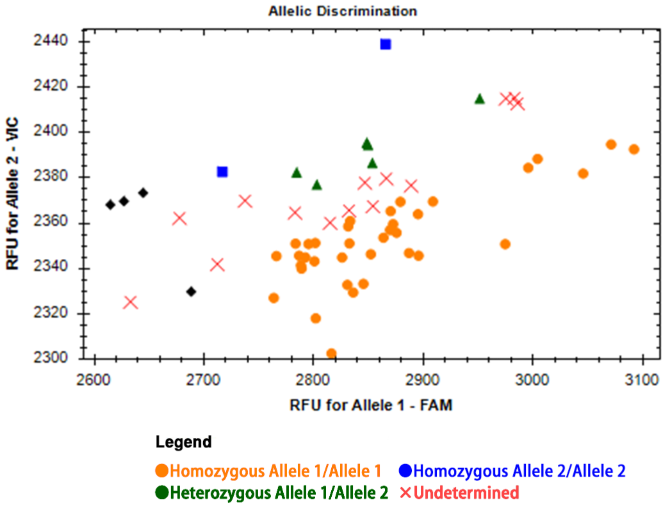

KASP-SNP28

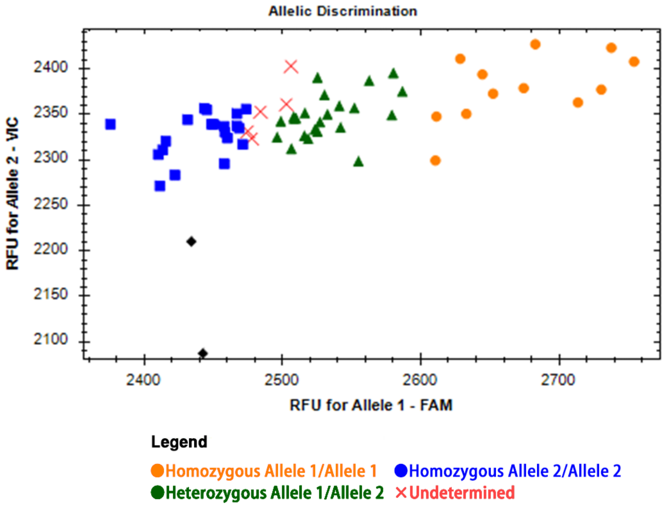

KASP-SNP18
